# 4-Azatricyclo[5.2.2.02,6]undecane-3,5,8-triones as Potential Pharmacological Agents

**DOI:** 10.3390/molecules13081570

**Published:** 2008-08-06

**Authors:** Jerzy Kossakowski

**Affiliations:** 1 Department of Medical Chemistry, The Medical University of Warsaw, 3 Oczki Street, 02-007 Warsaw, Poland; Tel./Fax. +48(22)6280679; 2 Faculty of Chemistry, Maria Curie-Sklodowska University, 20-031 Lublin, Poland

**Keywords:** 4-Azatricyclo[5.2.2.0^2,6^]undecane-3,5,8-trione, arylpiperazine derivatives, crystal structure, anti-HIV-1 activity

## Abstract

A series of twenty six arylpiperazine and aminoalkanol derivatives of 4-aza-tricyclo[5.2.2.0^2,6^]undecane-3,5,8-trione have been prepared. The synthesized compounds were evaluated for their cytotoxicity and anti-HIV-1 activity in MT-4 cells.

## Introduction

Currently available drugs for the treatment of HIV virus are based on combination of two types of anti-HIV-1 agents: nucleoside reverse transcriptase inhibitors (RTIs) and protease inhibitors [1]. The RTIs can be divided into nucleoside (NI) and non-nucleoside RT inhibitors (NNRTI). Several non-nucleoside inhibitors have been described, including nevirapine, thiobenzimidazolone (TIBO) derivatives, pyridinone derivatives and the bis(heteroaryl)piperazines (BHAPs), such as delavirdine and atevirdine [2]. Another arylpiperazine, vicriviroc, is currently in Phase II clinical trials [3]. However, the application of these agents is limited by serious side effects and the emergence of resistant strains. The discovery of new BHAPs analogs is actively proceeding [4,5]. Moreover, arylpiperazine derivatives exhibit a wide range of other biological activities: antiviral [6,7], anticancer [8,9], antioxidative [10], antibacterial [11] and antiarrythmic [12,13]. Many compounds of this class show high affinity for α_1_-adrenergic [14], dopaminergic [15] and serotoninergic receptors [16,17].

This work is a continuation of our investigation in the field of long-chain arylpiperazines [19], in a group of 4-azatricyclo[5.2.2.0^2,6^]undecane-3,5,8-trione derivatives. The newly synthesized compounds were evaluated for their inhibitory effects against the HIV-1 multiplication in acutely infected MT-4 cells (investigations performed at the Dipartamento di Scienze e Tecnologie Biomediche, Universita di Cagliari, Monserrato, Italy).

## Results and Discussion

**Scheme 1:**
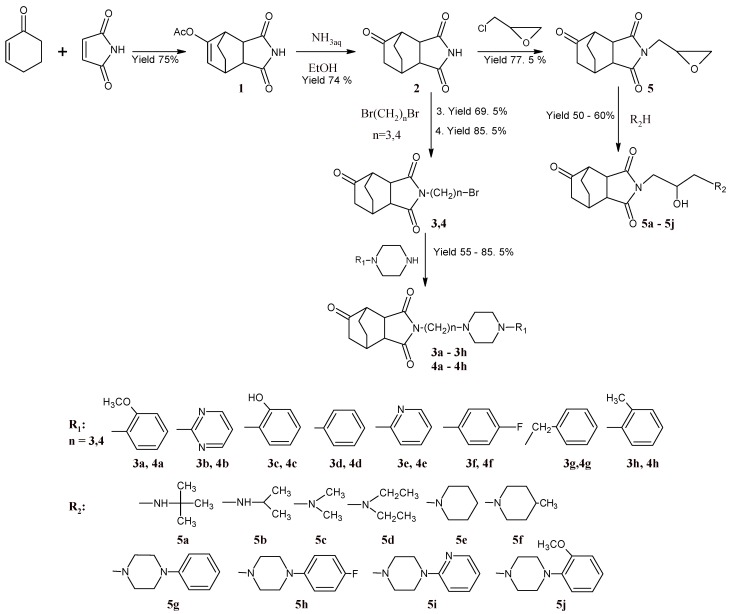
Synthesis of derivatives of 4-azatricyclo[5.2.2.0^2,6^]undecane-3,5,8-trione (**2**).

The first step of the multistage synthesis was the reaction of cyclohex-2-en-1-one with maleimide, in the presence of *p*-toluenosulfonic acid and isopropenyl acetate (Scheme 1). 3,5-Dioxo-4-aza-tricyclo[5.2.2.0^2,6^]undec-8-en-8-yl acetate (**1**) obtained in this reaction was then hydrolyzed by heating with aqueous-ethanolic solution of ammonia to give imide **2**. 

**Figure 1:**
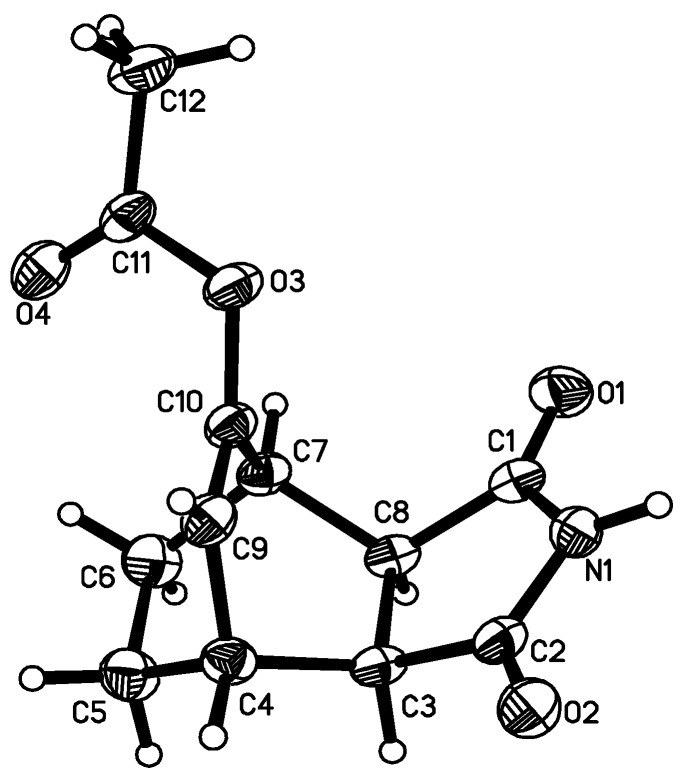
Molecular structure of starting compound **1**. The bond lengths within the imide fragment are: C1-O1 1.206(2), C1-N1 1.383(3), C2-O2 1.217(2), C2-N1 1.365(3), C1-C8 1.505(3), C2-C3 1.507(3), C3-C8 1.541(3) Å.

**Figure 2:**
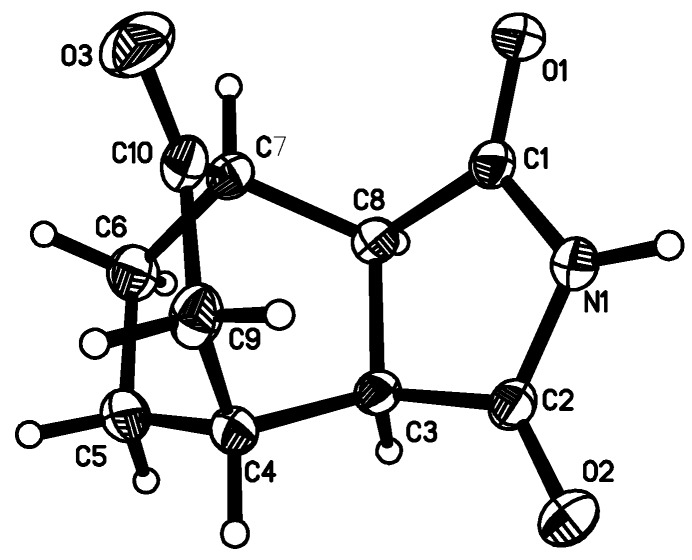
Molecular structure of starting compound **2**. The bond lengths within the imide fragment are: C1-O1 1.216(2), N1-C1 1.367(2), C2-O2 1.206(2), C2-N1 1.386(2), C2-C3 1.508(2), C1-C8 1.511(2), C8-C3 1.540(2) Å.

**Figure 3:**
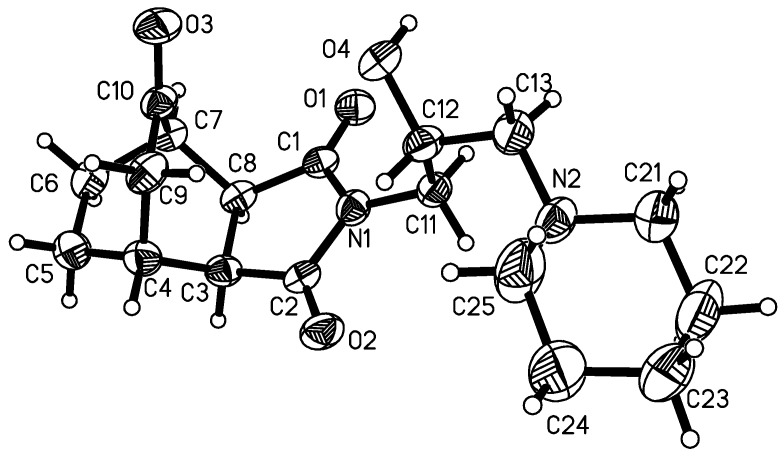
Molecular structure of product **5e**. Selected bond lengths: O1-C1 1.199(6), O2 C2 1.220(6), N1-C1 1.390(6), N1-C2 1.396(6), N1-C11 1.449(7), C2-C3 1.497(7), C3-C8 1.551(6), C1-C8 1.511(8) Å.

By alkylation of the latter with dibromoalkanes and 2-(chloromethyl)oxirane, the respective bromoalkyl- and 4-(oxiran-2-ylmethyl)- derivatives **3–5** were obtained. Next, the compounds were condensed with appropriate amines to give derivatives**3a–5j**. The general synthetic pathway is given in Scheme 1. The structure of all compounds have been established on the basis of elemental analysis, ^1^H-NMR and X-ray crystallography of **1**, **2** and **5e** (Figure 1, Figure 2 and Figure 3). 

The molecular geometry adopted in the solid-state by **1**, **2** and **5e** is influenced by a pattern of intermolecular contacts. The imide part of **1** and **2** is involved in intermolecular N-H···O hydrogen bonds which differentiate two peptide units: N1-C2=O2 and N1-C1=O1, causing the lengthening of the C=O bond and the shortening of C-N distance around the O atom, the latter being a hydrogen bond acceptor. The dimeric association around the center of symmetry is observed in the crystal structure of **1**, while molecules **2** form chains. In contrast, the imide moiety of **5e** is symmetric having equal respective bond lengths within the (O1)C1-N1-C2(O2) fragment. The hydrocarbon skeleton is rigid.

The molecular geometry adopted in the solid-state by **1**, **2** and **5e** is influenced by a pattern of intermolecular contacts. The imide part of **1** and **2** is involved in intermolecular N-H...O hydrogen bonds which differentiate two peptide units: N1-C2=O2 and N1-C1=O1, causing the lengthening of the C=O bond and the shortening of C-N distance around the O atom being an acceptor of hydrogen bond . The dimeric association around the center of symmetry is observed in the crystal structure of **1**, while molecules **2** form chains. In contrast, the imide moiety of **5e** is symmetric having equal respective bond lengths within the (O1)C1-N1-C2(O2) fragment. The hydrocarbon skeleton is rigid.

Thirty one new compounds were obtained. The synthesized compounds were evaluated for their cytotoxicity and anti-HIV-1 activity in MT-4 cells. The results are shown in Table 1. None of investigated compounds showed any anti HIV-1 activity, however, their cytotoxicity determined by the MTT method is greater then 100 μM.

**Table 1:** Cytotoxicity and anti-HIV activity of compounds **2−5j**.

*Compound*	^*a*^CC_50_	^*b*^EC_50_
	MT-4	HIV-1
**2, 3, 3a, 3b, 3c, 3d, 3f, 3h,** **4a, 4b, 4c, 4d, 4e, 4f, 4g, 4h,** **5a, 5b, 5c, 5d, 5e, 5f, 5g, 5h, 5i, 5j**	>100	>100

***^a^***Compound concentration (μM) required to reduce the viability of mock-infected MT-4 cells by 50%, as determined by the MTT method. ***^b^***Compound concentration (μM) required to achieve 50% protection of MT-4 cells from the HIV-1 induced cytopathogeneticy, as determined by the MTT method.

## Experimental

### General

All chemicals and solvents were purchased from Aldrich (Vienna, Austria). Melting points were determined on Electrothermal Digital Melting Point Apparatus (Essex, UK) and are uncorrected. The ^1^H-NMR spectra were recorded on a Bruker (Rheinstetten, Germany) spectrometer, operating at 400 MHz. The chemical shift values are expressed in ppm relative to TMS as an internal standard. Elemental analyses were recorded on a CHN model 2400 Perkin-Elmer (Hitachi, Tokyo, Japan). TLC was carried out using silica gel 60 F_254_, layer thickness 0.25 mm (E. Merck, Darmstadt, Germany) and the results were visualized using UV lamp at 254 nm. Column chromatography was carried out using silica gel 60 (200–400 mesh, Merck). The X-ray diffraction data were collected at 295 K with a KM4 diffractometer using graphite monochromated CuKα radiation (λ = 1.54178 Å) and ω/2θ scan mode. structures were solved by the SHELXS-97 program [20] and refined by full-matrix least-squares on *F*^2 ^using the SHELXL-97 program [21]. Non hydrogen atoms were refined with anisotropic displacement parameters. Carbon-bonded H-atoms were posititoned geometrically and ‘riding’ model was used in the refinement. The H-atoms of the hydroxyl, imide and piperidine groups were located on difference maps. The experimental details and final atomic parameters for **1**, **2** and **5e** have been deposited with the Cambridge Crystallographic Data Centre as supplementary material under deposition numbers CCDC 659529-659531, respectively. Copies of the data can be obtained free of charge on application to The Director, CCDC, 12 Union Road, Cambridge CB2 1EZ, UK, Fax: +44-1223-336-033; e-mail: deposit@ccdc.cam.ac.uk or www: http://www.ccdc.cam.ac.uk.

### Synthesis of 3,5-dioxo-4-azatricyclo[5.2.2.0^2,6^]undec-8-en-8-yl acetate (**1**)

A mixture of cyclohex-2-en-1-one (0.052 mol), maleimide (0.072 mol) and *p*-toluenesulfonic acid (50 mg) was refluxed for 6 h in isopropenyl acetate (15 cm^3^) The liquid was distilled off and the oily residue was crystallized from a hexane-ethyl acetate mixture (1:1) to give imide **1**. Yield 75%; m.p. 205–207°C; ^1^H-NMR (CDCl_3_) δ (ppm): 1.25–1.3 (m, 1H, CH_2_), 1.55–1.66 (m, 3H, CH_2_), 2.09 (s, 3H, CH_3_), 2.82–2.93 (m, 3H, CH-C=O, CH), 3.02 (d, 1H, *J* = 3.6 Hz, CH-C=O), 5.67 (d, 1H, *J* = 6.8 Hz, CH=), 11.08 (s, 1H, NH); Anal. Calcd. for C_12_H_13_NO_4_: C, 61.27, H, 5.57, N, 5.95. Found: C, 61.31, H, 5.69, N, 5.92; *Crystal data:* crystal system monoclinic, space group *P*2_1_/*n* with unit cell dimensions *a* = 8.662(2) Å, *b* = 11.884(2) Å, *c* = 10.754(2) Å, β= 96.22(3)°, *V* = 1100.5(4) Å^3^, Z = 4, *D*(*calcd*) = 1.420 g/cm^3^. Independent reflections 2234 [*R*(*int*) = 0.0194], number of parameters 155, final *R* indices [for 1412 reflections with *I* > 2σ(*I*)] *R*1 = 0.0396, *wR2* = 0.1086, and for all data *R*1 = 0.0842, *wR*2 = 0.1286. Largest residual peak and hole 0.15 and -0.22 e Å^-3^.

### Synthesis of 4-azatricyclo[5.2.2.0^2,6^]undecane-3,5,8-trione (**2**)

Imide **1** (0.043 mol) was refluxed for 1 h in anhydrous ethanol (80 mL) and 20% ammonia solution (15 mL). The liquid was filtered off and the residue was purified by crystallization from anhydrous ethanol to give compound **2. **Yield 74%; m.p. 223–224°C; ^1^H-NMR (CDCl_3_) δ (ppm): 1.68–1.94 (m, 5H, CH_2_, CH_2_-CH-CH_2_), 2.29 (d, 1H, *J* = 19.2 Hz, CH-C=O), 2.46–2.53 (m, 2H, CH_2_-C=O), 3.02 (dd, 1H, *J* = 2.3 Hz, CH-C=O), 3.21 (dd, 1H, *J* = 3.2 Hz, CH-C=O ), 11.35 (s, 1H, NH); Anal. Calcd. for C_10_H_11_NO_3_: C, 62.17, H, 5.72, N, 7.25. Found: C, 62.30, H, 5.8, N, 7.28; *Crystal data:* crystal system monoclinic, space group *P*2_1_/*c* with unit cell dimensions *a* = 9.941(2) Å, *b* = 10.628(2) Å, *c* = 8.300 (2) Å, β = 93.49 (3)°, *V* = 875.3(3) Å^3^, Z = 4, *D*(*calcd*) = 1.466 g/cm^3^. Independent reflections 1864 [*R*(*int*) = 0.0557], number of parameters 128, final *R* indices [for 1614 reflections with *I* > 2σ(*I*)] *R*1 = 0.0428, *wR2* = 0.1215, and for all data *R*1 = 0.0496, *wR*2 = 0.1267; extinction coefficient *x* = 0.020(2). Largest residual peak and hole 0.28 and -0.18 e Å^-3^. 

### General method for preparation of 4-(3-bromopropyl)- and 4-(4-bromobutyl)-4-aza-tricyclo[5.2.2.0^2,6^]undecane-3,5,8-trione (**3** and **4**)

A mixture of **2** (0.01 mol), 1,4-dibromobutane (0.03 mol) or 1,3-dibromopropane (0.03 mol) and anhydrous K_2_CO_3_ (0.014 mol) was dissolved in butanone (100 mL) and refluxed for 20 h. The solvent was distilled off and the oily residue was purified by column chromatography (eluting with chloroform) to give compounds **3** or **4**, respectively.

**3. **Yield 69.5%; m.p. 103–105°C; ^1^H-NMR (CDCl_3_) δ (ppm): 1.82 (d, 2H, *J* = 7.6 Hz, CH_2_), 1.95 (d, 2H, *J* = 6.8 Hz, CH_2_), 2.06–2.1 (m, 3H, CH-CH_2_, CH_2_), 2.24–2.29 (m, 1H, CH-CH_2_), 2.78 (d, 1H, *J* = 2.8 Hz, CH-C=O), 2.87 (d, 1H, *J* = 2.8 Hz, CH-C=O), 3.04 (dd, 1H, *J* = 3 Hz, CH_2_), 3.14 (dd, 1H, *J* = 4.1 Hz, CH_2_), 3.3−3.34 (m, 2H, CH_2_), 3.57–3.69 (m, 2H, CH_2_); Anal. Calcd. for C_13_H_16_NO_3_Br: C, 49.70, H, 5.13, N, 4.46. Found: C, 49.66, H, 5.14, N, 4.50.

**4**. Yield 85%; m.p. 84–86°C; ^1^H-NMR (CDCl_3_) δ (ppm): 1.66–1.68 (m, 2H, CH_2_), 1.73–1.84 (m, 4H, CH_2_), 1.95–1.98 (m, 2H, CH_2_), 2.06–2.11 (m, 1H, CH-CH_2_), 2.24–2.29 (m, 1H, CH-CH_2_), 2.78 (d, 1H, *J* = 2.4 Hz, CH_2_), 2.87 (d, 1H, *J* = 2.8 Hz, CH_2_), 3.03 (dd, 1H, *J* = 2.3 Hz, CH-C=O), 3.13 (dd, 1H, *J* = 3.1 Hz, CH-C=O), 3.4 (t, 2H, *J* = 6.2 Hz, CH_2_), 3.51 (t, 2H, *J* = 6.8 Hz, CH_2_); Anal. Calcd. for C_14_H_18_NO_3_Br·½ H_2_O: C, 49.86, H, 5.68, N, 4.12. Found: C, 49.91, H, 5.30, N, 4.12.

### General method for preparation of 4-substituted arylpiperazines with derivatives **3** and **4** (**3a–4h**)

A mixture of derivative **3** (0.012 mol) or **4** (0.016 mol), an appropriate amine (0.0024 or 0.0032 mol), anhydrous K_2_CO_3_ (0.003 mol) and catalytic amount of KI was dissolved in butanone (50 mL) and refluxed for 15 h. The solvent was evaporated, the residue was purified by column chromatography (eluting with chloroform-methanol 99.5:0.5) to give compounds **3a–3h** and **4a–4h**, respectively**.**

**3a. **Yield 80%; m.p. 215–217°C; ^1^H-NMR (CDCl_3_) δ (ppm): 1.82–1.83 (m, 4H, CH_2_), 1.94–1.97 (m, 2H, CH_2_), 2.08–2.12 (m, 1H, CH-CH_2_), 2.23–2.28 (m, 1H, CH-CH_2_ ), 2.42–2.56 (m, 6H, CH_2_), 2.77 (d, 1H, *J* = 2.8 Hz, CH-C=O), 2.87 (d, 1H, *J* = 2.8 Hz, CH-C=O), 3.03 (dd, 1H, *J* = 3.8 Hz, CH_2_), 3.14 (dd, 1H, *J* = 4.2 Hz, CH_2_), 3.38–3.45 (m, 6H, CH_2_), 3.76 (s, 3H, OCH_3_), 6.85–7.01 (m, 4H, CH_arom._), 10.88 (s, 1H, HCl); Anal. Calcd. for C_24_H_32_ClN_3_O_4_·H_2_O: C, 60.06, H, 7.14, N, 8.76. Found: C, 59.66, H, 7.40, N, 8.87.

**3b. **Yield 75%; m.p. 117–119°C; ^1^H-NMR (CDCl_3_) δ (ppm): 1.82–1.83 (m, 4H, CH_2_), 1.94–1.97 (m, 2H, CH_2_), 2.08–2.12 (m, 1H, CH-CH_2_), 2.23–2.28 (m, 1H, CH-CH_2_ ), 2.42–2.56 (m, 6H, CH_2_), 2.77 (d, 1H, *J* = 2.8 Hz, CH-C=O), 2.87 (d, 1H, *J* = 2.8 Hz, CH-C=O), 3.03 (dd, 1H, *J* = 3.8 Hz, CH_2_), 3.14 (dd, 1H, *J* = 4.2 Hz, CH_2_), 3.54 – 3.59 (m, 2H, CH_2_), 3.86–3.88 (m, 4H, CH_2_), 6.49 (t, 1H, *J* = 4.6 Hz, CH_arom.β_), 8.3 (d, 2H, J = 4.8 Hz, CH_arom.α_); Anal. Calcd. for C_21_H_27_N_5_O_3_·½ H_2_O: C, 62.05, H, 6.94, N, 17.23. Found: C, 62.16, H, 6.75, N, 16.86.

**3c. **Yield 70%; m.p. 250–252°C; ^1^H-NMR (CDCl_3_) δ (ppm): 1.74–1.81 (m, 4H, CH_2_), 1.92–1.93 (m, 2H, CH_2_), 2.08–2.12 (m, 1H, CH-CH_2_), 2.23–2.28 (m, 1H, CH-CH_2_ ), 2.42–2.56 (m, 6H, CH_2_), 2.77 (d, 1H, *J* = 2.8 Hz, CH-C=O), 2.87 (d, 1H, *J* = 2.8 Hz, CH-C=O), 3.03 (dd, 1H, *J* = 3.8 Hz, CH_2_), 3.14 (dd, 1H, *J* = 4.2 Hz, CH_2_), 3.38–3.44 (m, 6H, CH_2_), 6.73–6.89 (m, 4H, CH_arom._), 9.23 (s, 1H, OH), 10.45 (s, 1H, HCl); Anal. Calcd. for C_23_H_30_ClN_3_O_4_: C, 61.67, H, 6.75, N, 9.38. Found: C, 62.02, H, 6.72, N, 9.32.

**3d. **Yield 72%; m.p. 166–168°C; ^1^H-NMR (CDCl_3_) δ (ppm): 1.77–1.82 (m, 4H, CH_2_), 1.93–1.97 (m, 2H, CH_2_), 2.07–2.12 (m, 1H, CH-CH_2_), 2.22–2.27 (m, 1H, CH-CH_2_ ), 2.41 (t, 2H, *J* = 7 Hz, CH_2_), 2.6–2.61 (m, 4H, CH_2_), 2.76 (d, 1H, *J* = 2.8 Hz, CH-C=O), 2.86 (d, 1H, *J* = 2.8 Hz, CH-C=O), 3.0 (dd, 1H, *J* = 3.8 Hz, CH_2_), 3.11 (dd, 1H, *J* = 4.2 Hz, CH_2_), 3.19–3.22 (m, 4H, CH_2_), 3.57 (t, 2H, *J* = 7.2 Hz, CH_2_), 6.38–6.86 (m, 3H, CH_arom._), 7.23–7.27 (m, 2H, CH_arom._); Anal. Calcd. for C_23_H_29_N_3_O_3_: C, 69.85, H, 7.39, N, 10.62. Found: C, 69.34, H, 7.16, N,10.44.

**3e. **Yield 70%; m.p. 166–168°C; ^1^H-NMR (CDCl_3_) δ (ppm): 1.74–1.82 (m, 4H, CH_2_), 1.94–1.95 (m, 2H, CH_2_), 2.07–2.12 (m, 1H, CH-CH_2_), 2.23–2.28 (m, 1H, CH-CH_2_ ), 2.38 (t, 2H, *J* = 4.6 Hz, CH_2_), 2.53–2.55 (m, 4H, CH_2_), 2.76 (d, 1H, *J* = 2.8 Hz, CH-C=O), 2.86 (d, 1H, *J* = 2.8 Hz, CH-C=O), 3.01 (dd, 1H, *J* = 3.8 Hz, CH_2_), 3.11 (dd, 1H, *J* = 4.2 Hz, CH_2_), 3.53–3.59 (m, 6H, CH_2_), 6.6–6.65 (m, 2H, CH_arom._), 7.29–7.49 (m, 1H, CH_arom._), 8.17–8.18 (m, 1H, CH_arom._); Anal. Calcd. for C_22_H_28_N_4_O_3_·½H_2_O: C, 65.17, H, 7.21, N, 13.82. Found: C, 65.69, H, 6.84, N,13.71.

**3f. **Yield 73%; m.p. 125–127 °C; ^1^H-NMR (CDCl_3_) δ (ppm): 1.82–1.83 (m, 4H, CH_2_), 1.94–1.96 (m, 2H, CH_2_), 2.08–2.13 (m, 1H, CH-CH_2_), 2.24–2.29 (m, 1H, CH-CH_2_ ), 2.46 (m, 2H, CH_2_), 2.7–2.72 (m, 4H, CH_2_), 2.77 (d, 1H, *J* = 2.8 Hz, CH-C=O), 2.87 (d, 1H, *J* = 2.8 Hz, CH-C=O), 3.0 (dd, 1H, *J* = 3.8 Hz, CH_2_), 3.11 (dd, 1H, *J* = 4.2 Hz, CH_2_), 3.14–3.18 (m, 4H, CH_2_), 3.57 (t, 2H, *J* = 7.2 Hz, CH_2_), 6.85–6.97 (m, 4H, CH_arom._); Anal. Calcd. for C_23_H_28_FN_3_O_3_·2H_2_O: C, 61.46, H, 7.18, N, 9.35. Found: C, 61.70, H, 6.80, N, 9.22.

**3g. **Yield 65%; m.p. 135–137 °C; ^1^H-NMR (CDCl_3_) δ (ppm): 1.71–1.82 (m, 4H, CH_2_), 1.93–1.96 (m, 2H, CH_2_), 2.05–2.1 (m, 1H, CH-CH_2_), 2.22–2.27 (m, 1H, CH-CH_2_ ), 2.41 (t, 2H, *J* = 7 Hz, CH_2_), 2.57–2.61 (m, 5H, CH_2_), 2.75 (d, 1H, *J* = 2.8 Hz, CH-C=O), 2.85 (d, 1H, *J* = 2.8 Hz, CH-C=O), 3.0 (dd, 1H, *J* = 3.8 Hz, CH_2_), 3.11 (dd, 1H, *J* = 4.2 Hz, CH_2_), 3.19–3.22 (m, 4H, CH_2_), 3.46–3.67 (m, 4H, CH_2_), 7.29–7.49 (m, 5H, CH_arom._); Anal. Calcd. for C_24_H_32_ClN_3_O_3_·H_2_O: C, 62.13, H, 7.39, N, 9.06. Found: C, 62.52, H, 7.18, N, 8.89.

**3h. **Yield 70%; m.p. 115–117 °C; ^1^H-NMR (CDCl_3_) δ (ppm): 1.76–1.83 (m, 4H, CH_2_), 1.94–1.97 (m, 2H, CH_2_), 2.08–2.13 (m, 1H, CH-CH_2_), 2.24–2.27 (m, 1H, CH-CH_2_ ), 2.28 (s, 3H, CH_3_), 2.43 (t, 2H, *J* = 7 Hz, CH_2_), 2.6–2.64 (m, 4H, CH_2_), 2.76 (d, 1H, *J* = 2.8 Hz, CH-C=O), 2.87 (d, 1H, *J* = 2.8 Hz, CH-C=O), 3.93–3.96 (m, 4H, CH_2_), 3.0 (dd, 1H, *J* = 3.8 Hz, CH_2_), 3.13 (dd, 1H, *J* = 4.2 Hz, CH_2_), 3.55−3.59 (m, 2H, CH_2_), 6.95–7.02 (m, 2H, CH_arom._), 7.13–7.17 (m, 2H, CH_arom._); Anal. Calcd. for C_24_H_31_N_3_O_3_: C, 70.39, H, 7.63, N, 10.26. Found: C, 69.99, H, 7.35, N, 10.02.

**4a. **Yield 80%; m.p. 204–206°C; ^1^H-NMR (CDCl_3_) δ (ppm): 1.47–1.54 (m, 2H, CH_2_), 1.81–1.83 (m, 2H, CH_2_), 1.94–1.97 (m, 2H, CH_2_), 2.06–2.11 (m, 1H, CH-CH_2_), 2.23–2.28 (m, 1H, CH-CH_2_), 2.48–2.5 (m, 2H, CH_2_), 2.73–2.77 (m, 6H, CH_2_), 2.87–2.88 (m, 1H, CH_2_), 3.0–3.03 (m, 1H, CH-C=O), 3.11–3.14 (m, 1H, CH-C=O), 3.14–3.16 (m, 6H, CH_2_), 3.49−3.51 (m, 2H, CH_2_), 3.86 (s, 3H, OCH_3_), 6.84–7.01 (m, 4H, CH_arom._); Anal. Calcd. for C25H34ClN3O4: C, 63.08, H, 7.20, N, 8.83. Found: C, 63.53, H, 7.23, N, 8.87.

**4b. **Yield 78%; m.p. 139–140°C; ^1^H-NMR (CDCl_3_) δ (ppm): 1.47–1.55 (m, 4H, CH_2_), 1.82–1.83 (m, 2H, CH_2_), 1.94–1.98 (m, 2H, CH_2_), 2.06–2.11 (m, 1H, CH-CH_2_), 2.23–2.28 (m, 1H, CH-CH_2_), 2.35–2.39 (m, 2H, CH_2_), 2.46−2.49 (m, 4H, CH_2_), 2.77 (d, 1H, CH_2_), 2.87 (d, 1H, CH_2_), 3.0–3.02 (m, 1H, CH-C=O), 3.1–3.13 (m, 1H, CH-C=O), 3.48–3.52 (m, 2H, CH_2_), 3.8−3.82 (m, 4H, CH_2_), 6.47 (t, 1H, *J* = 4.8 Hz, CH_arom. β_), 8.3 (d, 2H, *J* = 4.8 Hz, CH_arom. α_); Anal. Calcd. for C_22_H_30_ClN_5_O_3_·½H_2_O: C, 62.84, H, 7.19, N, 16.66. Found: C, 62.82, H, 6.89, N, 16.37.

**4c. **Yield 65%; m.p. 243–245 °C; ^1^H-NMR (CDCl_3_) δ (ppm): 1.47–1.53 (m, 4H, CH_2_), 1.63–1.85 (m, 2H, CH_2_), 1.8–1.82 (m, 2H, CH_2_), 2.12–2.17 (m, 1H, CH-CH_2_), 2.24–2.29 (m, 1H, CH-CH_2_), 2.4–2.41 (m, 2H, CH_2_), 2.56 (m, 3H, CH_2_,), 2.9−2.92 (m, 1H, CH_2_), 3.0–3.01 (m, 4H, CH_2_), 3.0–3.01 (m, 1H, CH-C=O), 3.02–3.03 (m, 1H, CH-C=O), 3.5–3.51 (m, 4H, CH_2_), 6.85–7.18 (m, 4H, CH_arom._); Anal. Calcd. for C_24_H_32_ClN_3_O_4_: C, 62.39, H, 6.98, N, 9.10. Found: C, 62.50, H, 6.21, N, 8.89.

**4d. **Yield 67%; m.p. 200–202°C; ^1^H-NMR (CDCl_3_) δ (ppm): 1.48–1.49 (m, 4H, CH_2_), 1.72–1.8 (m, 2H, CH_2_), 1.81–1.83 (m, 2H, CH_2_), 2.06–2.11 (m, 1H, CH-CH_2_), 2.23–2.28 (m, 1H, CH-CH_2_), 2.38–2.39 (m, 2H, CH_2_), 2.56–2.58 (m, 4H, CH_2_,), 2.77 (d, 1H, CH_2_), 2.87 (d, 1H, CH_2_), 2.97–3.01 (m, 1H, CH-C=O), 3.09–3.12 (m, 1H, CH-C=O), 3.19–3.2 (m, 4H, CH_2_), 2.51–3.53 (m, 2H, CH_2_), 6.84–6.93 (m, 5H, CH_arom._); Anal. Calcd. for C_24_H_33_Cl_2_N_3_O_3_·½H_2_O: C, 58.66, H, 6.97, N, 8.55. Found: C, 58.88, H, 6.76, N, 8.36.

**4e. **Yield 72%; m.p. 129–130°C; ^1^H-NMR (CDCl_3_) δ (ppm): 1.48–1.54 (m, 4H, CH_2_), 1.75–1.83 (m, 2H, CH_2_), 1.94–1.96 (m, 2H, CH_2_), 2.06–2.11 (m, 1H, CH-CH_2_), 2.24–2.29 (m, 1H, CH-CH_2_), 2.36–2.39 (m, 2H, CH_2_), 2.53–2.54 (m, 4H, CH_2_,), 2.77 (d, 1H, CH_2_), 2.88 (d, 1H,CH_2_), 2.99–3.01 (m, 1H, CH-C=O), 3.09–3.12 (m, 1H, CH-C=O), 3.49–3.53 (m, 6H, CH_2_), 6.59–6.64 (m, 2H, CH_arom._), 7.46 (t, 1H, *J* = 7.2 Hz, CH_arom.γ_), 8.16 (d, 1H, *J* = 3.6 Hz, CH_arom.α_); Anal. Calcd. for C_23_H_30_N_4_O_3_: C, 67.29, H, 7.37, N, 13.65. Found: C, 66.92, H, 7.16, N, 13.46.

**4f. **Yield 70%; m.p. 72–74°C; ^1^H-NMR (CDCl_3_) δ (ppm): 1.47–1.53 (m, 4H, CH_2_), 1.82–1.93 (m, 2H, CH_2_), 1.94–1.95 (m, 2H, CH_2_), 2.05–2.1 (m, 1H, CH-CH_2_), 2.22–2.27 (m, 1H, CH-CH_2_), 2.37–2.4 (m, 2H, CH_2_), 2.59–2.6 (m, 4H, CH_2_,), 2.87 (d, 1H, CH_2_), 2.99 (d, 1H, CH_2_), 3.01–3.09 (m, 1H, CH-C=O), 3.11–3.12 (m, 3H, CH_2_, CH-C=O), 3.48–3.51 (m, 2H, CH_2_), 6.86–6.96 (m, 4H, CH_arom._); Anal. Calcd. for C_24_H_31_ClFN_3_O_3_: C, 62.13, H, 6.73, N, 9.06. Found: C, 62.09, H, 6.75, N, 8.94.

**4g. **Yield 60%; m.p. 175–177°C; ^1^H-NMR (CDCl_3_) δ (ppm): 1.42–1.51 (m, 4H, CH_2_), 1.8–1.82 (m, 2H, CH_2_), 1.93–1.95 (m, 2H, CH_2_), 2.04–2.09 (m, 1H, CH-CH_2_), 2.21–2.26 (m, 1H, CH-CH_2_), 2.3–2.34 (m, 2H, CH_2_), 2.24–2.49 (m, 8H, CH_2_,), 2.75 (d, 1H, *J* = 2.4 Hz, CH_2_), 2.85 (d, 1H, *J* = 2.8 Hz, CH_2_), 2.97–3.0 (m, 1H, CH-C=O), 3.08–3.11 (m, 1H, CH-C=O), 3.45–3.49 (m, 4H, CH_2_), 7.23–7.3 (m, 5H, CH_arom._); Anal. Calcd. for C_25_H_34_ClN_3_O_3_·3H_2_O: C, 58.41, H, 7.84, N, 8.18. Found: C, 58.63, H, 7.91, N 8.37.

**4h. **Yield 70%,m.p. 109–110°C, ^1^H-NMR (CDCl_3_) δ (ppm): 1.48–1.54 (m, 4H, CH_2_), 1.81–1.83 (m, 2H, CH_2_), 1.94–1.96 (m, 2H, CH_2_), 2.07–2.12 (m, 1H, CH-CH_2_), 2.13–2.28 (m, 1H, CH-CH_2_), 2,29 (s, 3H, CH_3_), 2.38–2.42 (m, 2H, CH_2_), 2.56–2.58 (m, 4H, CH_2_,), 2.77 (d, 1H, *J* = 2.4 Hz, CH_2_), 2.88 (d, 1H, *J* = 2.4 Hz, CH_2_), 2.92–2.93 (m, 4H, CH_2_), 3.01–3.02 (m, 1H, CH-C=O), 3.12–3.13 (m, 1H, CH-C=O), 3.49–3.52 (m, 2H, CH_2_), 6.95–7.17 (m, 4H, CH_arom._); Anal. Calcd. for C_25_H_33_N_4_O_3_: C, 70.89, H, 7.85, N, 9.92. Found: C, 70.60, H, 7.62, N, 9.82.

### Synthesis of 4-(oxiran-2-ylmethyl)-4-azatricyclo[5.2.2.0^2,6^]undecane-3,5,8-trione (**5**)

A mixture of imide **2 **(0.01 mol), 2-(chloromethyl)oxirane (26 mL) and anhydrous K_2_CO_3_ (0.01 mol) was refluxed on water bath for 30 h. The solvent was distilled off, then the oily residue was purified by column chromatography (chloroform-methanol 99.5:0.5). Yield 77.5%; m.p. 172–173.5°C; ^1^H-NMR (CDCl_3_) δ (ppm): 1.82–1.84 (m, 2H, CH_2_), 1.95–1.98 (m, 2H, CH_2_), 2.21–2.25 (m, 2H, CH_2_), 2.45–2.67 (m, 1H, CH), 2.72 (t, 1H, *J* = 4.2 Hz, CH-C=O), 2.77 (d, 1H, *J* = 4 Hz, CH-C=O), 2.89 (d, 1H, J = 3.2 Hz, CH-C=O), 3.05–3.13 (m, 2H, CH_2_), 3.16–3.19 (m, 1H, CH-O), 3.58–3.81 (m, 2H, CH_2_); Anal. Calcd. for C_13_H_15_NO_4_·½H_2_O: C, 60.46, H, 6.24, N, 5.42. Found: C, 59.95, H, 5.79, N 5.14.

### General method for preparation of 4-(amino)-2-hydroxypropyl derivatives of 4-aza-tricyclo[5.2.2.0^2,6^]undecane-3,5,8-trione **5a–5j**

A mixture of **5** (0.001 mol), an appropriate amine (0.0015 mol) and water (1 mL) was dissolved in methanol (40 mL) and heated on water bath in 75°C for 20 h. The liquid was distilled off, the oily residue was purified by column chromatography (chloroform-methanol 99.5:0.5) to give compounds **5a–5j**.

**5a. **Yield 60%; m.p. 260–262°C; ^1^H-NMR (CDCl_3_) δ (ppm): 1.28 (s, 9H, CH_3_), 1.68–1.78 (m, 2H, CH_2_), 1.94–1.98 (m, 2H, CH_2_), 2.24–2.26 (m, 2H, CH_2_), 2.43–2.53 (m, 2H, CH_2_), 2.74–2.8 (m, 1H, CH-C=O), 2.82–2.88 (m, 2H, CH-C=O), 3.06–3.2 (m, 2H, CH_2_), 3.51–3.79 (m, 2H, CH_2_), 3.87–3.88 (m, 1H, CH-OH), 5.67 (s, 1H, OH), 8.49 (s, 1H, NH), 8.99 (s, 1H, HCl); Anal. Calcd. for C_17_H_27_ClN_2_O_4_: C, 56.90, H, 7.58, N, 7.81. Found: C, 56.47, H, 7.37, N, 7.65.

**5b. **Yield 65%; m.p. 206–208°C; ^1^H-NMR (CDCl_3_) δ (ppm): 1.09 (d, 6H, *J* = 6 Hz, CH_3_); 1.8–1.83 (m, 2H, CH_2_); 1.94–1.98 (m, 2H, CH_2_); 2.24–2.26 (m, 2H, CH_2_); 2.43–2.53 (m, 3H, CH_2_, CH); 2.74–2.8 (m, 1H, CH-C=O); 2.82–2.88 (m, 3H, CH, CH-C=O); 3.06–3.2 (m, 2H, CH_2_), 3.51–3.79 (m, 2H, CH_2_); 3.87–3.88 (m, 1H, CH-OH); Anal. Calcd. for C_16_H_25_ClN_2_O_4_: C, 55.73; H, 7.31; N, 8.12. Found: C, 55.30; H, 7.15; N, 7.84.

**5c. **Yield 60%; m.p. 72–74°C; ^1^H-NMR (CDCl_3_) δ (ppm): 1.28 (s, 9H, CH_3_); 1.67–1.76 (m, 2H, CH_2_); 1.81–2.05 (m, 2H, CH_2_); 2.24–2.26 (m, 2H, CH_2_); 2.43–2.53 (m, 3H, CH_2_, CH); 2.47 (s, 6H, CH_3_); 2.74–2.8 (m, 1H, CH-C=O); 2.82–2.88 (m, 2H, CH-C=O); 3.06–3.2 (m, 2H, CH_2_), 3.51–3.79 (m, 2H, CH_2_); 3.87–3.88 (m, 1H, CH-OH); Anal. Calcd. for C_15_H_23_ClN_2_O_4_·1½H_2_O: C, 50.35; H, 7.32; N, 7.83. Found: C, 50.05; H, 7.00; N, 7.49.

**5d. **Yield 72%; m.p. 121–123°C; ^1^H-NMR (CDCl_3_) δ (ppm): 1.06–1.14 (m, 6H, CH_3_), 1.8–1.82 (m, 2H, CH_2_), 1.94–1.97 (m, 2H, CH_2_), 2.24–2.26 (m, 2H, CH_2_), 2.43–2.53 (m, 3H, CH_2_, CH), 2.74–2.8 (m, 5H, CH_2_, CH-C=O), 2.82–2.88 (m, 2H, CH-C=O), 3.06–3.17 (m, 2H, CH_2_), 3.52–3.75 (m, 2H, CH_2_), 3.86–3.84 (m, 1H, CH-OH); Anal. Calcd. for C_17_H_26_N_2_O_4_·^1^/_3_H_2_O: C, 62.17, H, 8.18, N, 8.53. Found: C, 62.40, H, 7.87, N, 8.41.

**5e. **Yield 75.5%; m.p. 132–134°C; ^1^H-NMR (CDCl_3_) δ (ppm): 1.47–1.49 (m, 2H, CH_2_), 1.66−1.69 (m, 4H, CH_2_), 1.8–1.83 (m, 2H, CH_2_), 1.95–1.97 (m, 2H, CH_2_), 2.24–2.26 (m, 2H, CH_2_), 2.43–2.53 (m, 5H, CH_2_, CH), 2.74–2.8 (m, 1H, CH-C=O), 2.72–2.87 (m, 3H, CH_2_, CH-C=O), 3.05–3.17 (m, 2H, CH_2_), 3.51–3.79 (m, 2H, CH_2_), 4.13–3.91 (m, 1H, CH-OH); Anal. Calcd. for C_18_H_26_N_2_O_4_: C, 64.65, H, 7.84, N, 8.38. Found: C, 64.35, H, 7.62, N, 8.32; *Crystal data:* crystal system monoclinic, space group *Pc* with unit cell dimensions *a* = 12.496(2) Å, *b* = 6.242(1) Å, *c* = 11.351(2) Å, β = 94.36 (3)°, *V* = 882.8(3) Å^3^, Z = 2, *D*(*calcd*) = 1.258 g/cm^3^. Independent reflections 1852, number of parameters 215, final *R* indices [for 986 reflections with *I* > 2σ(*I*)] *R*1 = 0.0601, *wR2* = 0.1336, and for all data *R*1 = 0.1361, *wR*2 = 0.1656. Largest residual peak and hole 0.31 and -0.30 e Å^-3^. 

**5f. **Yield 75.5%; m.p. 120–122°C; ^1^H-NMR (CDCl_3_) δ (ppm): 0.91 (d, 3H, J = 6.4 Hz, CH_3_), 1.23–1.39 (m, 3H, CH_2_, CH), 1.57–1.65 (m, 2H, CH_2_), 1.8–1.83 (m, 2H, CH_2_), 1.94–1.96 (m, 2H, CH_2_), 2.24–2.26 (m, 2H, CH_2_), 2.33–2.44 (m, 2H, CH_2_), 2.43–2.53 (m, 3H, CH_2_, CH), 2.74–2.8 (m, 1H, CH-C=O), 2.82–2.88 (m, 3H, CH, CH-C=O), 3.06–3.2 (m, 2H, CH_2_), 3.51–3.79 (m, 2H, CH_2_), 3.87–3.88 (m, 1H, CH-OH); Anal. Calcd. for C_19_H_28_N_2_O_4_: C, 65.49, H, 8.10, N, 8.04. Found: C, 65.39, H, 7.91, N, 7.99.

**5g. **Yield 70%; m.p. 186−188°C; ^1^H-NMR (CDCl_3_) δ (ppm): 1.66–1.79 (m, 2H, CH_2_), 1.98–2.05 (m, 3H, CH, CH_2_), 2.19–2.24 (m, 1H, CH-C=O), 2.47–2.56 (m, 2H, CH_2_), 3.11–3.15 (m, 6H, CH_2_), 3.29–3.58 (m, 2H, CH-C=O), 3.6–3.75 (m, 6H, CH_2_), 4.21–4.24 (m, 1H, CH-OH), 6.82 (t, 1H, *J* = 7.2 Hz, CH_arom._), 6.94 (d, 2H, *J* = 8.4 Hz, CH_arom._), 7.22 (t, 2H, *J* = 7.8 Hz, CH_arom._), 10.67 (s, 1H, HCl); Anal. Calcd. for C_23_H_30_ClN_3_O_4_: C, 61.67, H, 6.75, N, 9.38. Found: C, 61.39, H, 6.50, N, 9.16.

**5h. **Yield 80%; m.p. 211–213°C; ^1^H-NMR (CDCl_3_) δ (ppm): 1.78–1.82 (m, 2H, CH_2_), 1.95–1.98 (m, 3H, CH, CH_2_), 2.21–2.57 (m, 3H, CH-C=O, CH_2_), 2.74–2.88 (m, 6H, CH_2_), 3.07–3.16 (m, 6H, CH-C=O, CH_2_), 3.58–3.63 (m, 2H, CH_2_), 4.09 (m, 1H, CH-OH), 6.86–6.95 (m, 4H, CH_arom._); Anal. Calcd. for C_23_H_28_N_3_O_4_F: C, 64.32, H, 6.57, N, 9.79. Found: C, 63.94, H, 6.30, N, 9.51.

**5i. **Yield 75%; m.p. 153–155°C; ^1^H-NMR (CDCl_3_) δ (ppm): 1.82–1.84 (m, 2H, CH_2_), 1.94–1.97 (m, 2H, CH_2_), 2.28–2.32 (m, 1H, CH), 2.35–2.43 (m, 3H, CH-C=O, CH_2_), 2.58–2.88 (m, 6H, CH_2_), 3.06 (d, 1H, *J* = 8.8 Hz, CH-C=O), 3.16 (d, 1H, *J* = 8.8 Hz, CH-C=O), 3.53–3.58 (m, 6H, CH_2_), 3.95–4.05 (m, 1H, CH-OH), 6.25 (m, 2H, CH_arom._), 7.47 (t, 1H, *J* = 7.4 Hz, CH_arom._), 8.18 (d, 1H, *J* = 4 Hz, CH_arom._); Anal. Calcd. for C_22_H_28_N_4_O_4_·½H_2_O: C, 62.69, H, 6.93, N, 13.29. Found: C, 62.92, H, 6.54, N, 12.88.

**5j. **Yield 75%; m.p. 147–149°C; ^1^H-NMR (CDCl_3_) δ (ppm): 1.81–1.83 (m, 2H, CH_2_), 1.96–1.98 (m, 2H, CH_2_), 2.2–2,37 (m, 2H, CH, CH-C=O), 2.55–2.66 (m, 2H, CH_2_), 2.77–3.19 (m, 12H, CH_2_, CH-C=O), 3.6–3.75 (m, 2H, CH_2_), 3.85 (s, 3H, OCH_3_), 4.08–4.24 (m, 1H, CH-OH), 6.82–7.01 (m, 4H, CH_arom._); Anal. Calcd. for C_24_H_31_N_3_O_5_: C, 65.29, H, 7.08, N, 9.52. Found: C, 65.26, H, 6.76, N, 9.12.

### Biological Assays: Compounds

Compounds were dissolved in DMSO at 100 mM and then diluted in the culture medium.

### Cells and Viruses

Cell lines were purchased from American Type Culture Collection (ATCC). The absence of mycoplasma contamination was checked periodically by the Hoechst staining method. Cell lines supporting the multiplication of RNA viruses were the following: CD4^+^ human T-cells containing an integrated HTLV-1 genome (MT-4).

### Cytotoxicity Assays

For cytotoxicity evaluations, exponentially growing cells derived from human haematological tumors [CD4^+^ human T-cells containing an integrated HTLV-1 genome (MT-4)] were seeded at an initial density of 1×10^5^ cells/mL in 96 well plates in RPMI-1640 medium supplemented with 10% fetal calf serum (FCS), 100 units/mL penicillin G and 100 µg/mL streptomycin. Cell cultures were then incubated at 37°C in a humidified, 5% CO_2_ atmosphere in the absence or presence of serial dilutions of test compounds. Cell viability was determined after 96 hrs at 37°C by the 3-(4,5-dimethylthiazol-2-yl)-2,5-diphenyl-tetrazolium bromide (MTT) method [22].

### Antiviral assay

Activity of compounds against Human Immunodeficiency virus type-1 (HIV-1) was based on inhibition of virus-induced cytopathogenicity in MT-4 cells acutely infected with a multiplicity of infection (m.o.i.) of 0.01. Briefly, 50 μL of RPMI containing 2×10^4^ MT-4 were added to each well of flat-bottom microtitre trays containing 50 μL of RPMI, without or with serial dilutions of test compounds. Then, 20 μL of an HIV-1 suspension containing 100 CCID_50_ were added. After a 4-day incubation, cell viability was determined by the MTT method. 
